# Down-regulation of Human Leukocyte Antigen class I heavy chain in tumors is associated with a poor prognosis in advanced esophageal cancer patients

**DOI:** 10.3892/ijo.2011.1274

**Published:** 2011-11-30

**Authors:** KIMITAKA TANAKA, TAKAHIRO TSUCHIKAWA, MASAKI MIYAMOTO, TAKEHIRO MAKI, MASAOMI ICHINOKAWA, KANAKO C. KUBOTA, TOSHIAKI SHICHINOHE, SATOSHI HIRANO, SOLDANO FERRONE, HIROTOSHI DOSAKA-AKITA, YOSHIHIRO MATSUNO, SATOSHI KONDO

**Affiliations:** 1 Department of Surgical Oncology, Hokkaido University Graduate School of Medicine, Sapporo, Japan; 2 Department of Surgical Pathology, Hokkaido University Graduate School of Medicine, Sapporo, Japan; 3 Departments of Surgery, Immunology and Pathology, University of Pittsburgh Cancer Institute, Pittsburgh, PA, USA; 4 Department of Medical Oncology, Hokkaido University Graduate School of Medicine, Sapporo, Japan

**Keywords:** histocompatibility antigens class I, antigen processing machinery, esophageal neoplasms, tissue array analysis, immunohistochemistry

## Abstract

The HLA class I antigen processing machinery (APM) plays a crucial role in the anticancer immune response. The aim of this study was to assess the clinical significance of APM components in esophageal cancer. A total of 11 esophageal cancer cell lines were evaluated by Western blot analysis for 13 HLA class I APM components. There was a different expression pattern among cancer cell lines for HLA class I heavy chain (HLA-HC), β2 microglobulin, Tapasin, TAP-1, TAP-2, LMP-7 and LMP-10. Immunohistochemical staining utilizing a tissue microarray method for HLA class I APM expression showing different expression patterns among cell lines was performed for 95 surgical specimens from patients with esophageal cancer. Prognostic factors were the down-regulation of HLA-HC, and the up-regulation of β2 microglobulin and TAP-1 in the cancer tissues. Multivariate analysis using a Cox regression model indicated that the down-regulation of HLA-HC, and up-regulation of TAP-1 in cancer tissues are independent, unfavorable prognostic factors (hazard ratio, 2.361 and 2.297; P=0.0141 and 0.0145, respectively). Although there was no significant difference in survival for selected p-stage I and II patients (n=54) in all APM components, only down-regulation of HLA-HC was an unfavorable prognostic factor by a Cox regression model for selected p-stage III and IV patients (n=41). In conclusion, the current results suggest that the down-regulation of HLA-HC in tumors is especially associated with a poor prognosis among advanced esophageal cancer patients.

## Introduction

Esophageal cancer is a malignant disease with a 5-year survival after esophagectomy of around 50% according to the report of the Japan Clinical Oncology Group (1). Although advances in diagnosis and treatment of esophageal squamous cell carcinoma (ESCC) have been made in recent years, postoperative survival rates have not improved in the last decade (2–4). Therefore, new clinical parameters for prognosis and new approaches for adjuvant treatment are needed. Cancer-specific immunotherapy is considered to be a new therapeutic modality. We have been investigating tumor microenvironments that affect patient survival. In previous studies, we found that cooperation between CD4^+^ and CD8^+^ T cells appears to improve the prognosis of ESCC patients (5). Thus, the host immune response against cancer cells and immune escape mechanisms by the tumor (6) seem to play crucial roles in the prevention of disease recurrence and determine the postoperative prognosis in ESCC.

Human leukocyte antigen (HLA) class I molecules are critical for the presentation of antigen peptides derived from tumor cells to cytotoxic T lymphocytes (CTLs). Antigen processing machinery (APM) is the combination of cellular processes responsible for the presentation of endogenous peptides by HLA class I molecules. APM is essential for the successful presentation of HLA class I antigens. Loss of surface-expressed HLA class I molecules is particularly important for cancer cell proliferation and metastasis, because this enables tumor cells to evade recognition and lysis by CTLs (7–10). Therefore, down-regulation of APM components may lead to defects in the expression of HLA class I-peptide complexes and eventually enable tumor cells to escape from the host immunosurveillance mediated by CTLs (11–13).

The association between down-regulation of several APM components and cancer prognosis has been reported in a wide range of malignancies, including lung, breast, uterine cervix, head and neck area, larynx, ovary, kidney, skin, and ESCC (14–28). To the best of our knowledge, there have been few reports that have comprehensively analyzed the correlations between HLA class I pathway expression and patient prognosis in ESCC. Here, the expressions of HLA class I heavy chain (HLA-HC), β2 microglobulin, and 11 APM components are reported in various esophageal cell lines. There was a correlation between several APM components and esophageal cancer prognosis by tissue microarray method.

## Materials and methods

### Cell lines

The ESCC cell lines TE2, TE4, TE5, TE6, TE8, TE9, TE10, TE13, TE14, HEC46, and SGF7, and the lung adenocarcinoma cell line LCD were used. Human squamous cell carcinoma of esophagus cell line TE series was generously provided by Dr T. Nishihira (University of Tohoku, Japan). HEC46 was provided by Dr T. Toge (University of Hiroshima, Japan), and SGF7 was provided by Dr T. Saito (Toyama Medical and Pharmaceutical University, Japan). LCD was obtained from the Japanese Cancer Research Resources Bank (Tokyo, Japan). All cell lines were grown in RPMI-1640 (Sigma-Aldrich Japan, Tokyo, Japan) with 10% fetal bovine serum (FBS) and 1% penicillin/streptomycin, and they were maintained in a humidified incubator with 5% CO_2_ in air at 37˚C.

### Mice and xenograft models

CB17/severe combined immunodeficiency (SCID) mice were obtained from Charles River Japan (Yokohama, Japan). All mice were female, aged 6 to 8 weeks, and maintained under specific pathogen-free conditions. All animal procedures were conducted in accordance with the guidelines of the Hokkaido University Institutional Animal Care and Use Committee. Cultured esophageal and lung cancer cells (over 5×10^6^) were injected subcutaneously with a volume of 100 μl of phosphate-buffered saline (PBS) into the right flank region of each CB17/SCID mouse. When tumor size exceeded 15 mm in diameter, the mice were euthanized, and the resected tumors were separated into two blocks: one block was frozen in liquid nitrogen to extract proteins for Western blot analysis, and the other was immersed in formalin for immunohistologic analysis.

### Antibodies

The mouse monoclonal antibody (mAb) EMR8-5, which recognizes the heavy chains of HLA-A, HLA-B, and HLA-C, was purchased from Hokudo Co., Ltd. (Sapporo, Japan). The β2 microglobulin-specific mAb NAMB-1, tapasin-specific mAb TO-3, calnexin-specific mAb TO-5, calreticulin-specific mAb TO-11, ERp57-specific mAb TO-2, TAP-1-specific mAb NOB-1, TAP-2-specific mAb NOB-2, LMP-7-specific mAb HB-2, LMP-10-specific mAb TO-7, MB-1-specific mAb SY-5, Delta-specific mAb SJJ-3, and Z-specific mAb NB-1 were established and characterized at the Department of Immunology, Rosewell Park Cancer Institute (Buffalo, NY) (29,30). Anti-β actin mouse monoclonal antibody (MAB1501R) was purchased from Millipore (Tokyo, Japan). Negative control mouse IgG2b (X0943) and IgG1 (X0931) were purchased from Dako Japan (Kyoto, Japan).

Peroxidase-conjugated AffiniPure goat anti-mouse IgG (H+L) was purchased from Jackson ImmunoResearch (West Grove, PA).

### Western blot analysis

Western blotting was performed following the methodology previously described with minor modifications (30). In brief, lysates from cell lines, CB17/SCID mouse xenografts, and normal human esophageal/lung tissue were mixed with SDS-PAGE sample buffer, boiled for 3 min, and then separated on 10–15% polyacrylamide gels. Separated proteins (20 μg/lane) were transferred to nitrocellulose membranes (Amersham, Tokyo, Japan) or PVDF membranes (Amersham). After blocking with 1 h incubation in PBS containing 2% BSA and 5% non-fat dry milk, the membrane was incubated overnight at 4˚C with antibodies (1:200 in 1% dry milk/TBS-T). The membrane was washed three times for 5 min each in PBS containing 0.1% Tween 20 and incubated for 1 h at room temperature with peroxidase-conjugated goat anti-mouse IgG, Fc fragment antibody in PBS containing 1% non-fat dry milk/TBS-T. Three additional washings for 5 min each in PBS containing 0.1% Tween 20 followed. The detection binding antibodies were performed using the ECL Plus system (Amersham). Lysates from normal human esophageal mucosa and normal human lung tissue were used as positive controls.

### Immunohistochemistry

Immunohistochemical reactions were carried out using the universal immunoenzyme polymer method (Nichirei Corp, Tokyo, Japan). The mouse monoclonal primary antibodies used were EMR8-5, NAMB-1, TO-3, NOB-1, NOB-2, HB-2, and TO-7. Briefly, paraffin-embedded tissue sections were deparaffinized with xylene and rehydrated in a graded series of ethanol solutions. Antigens were retrieved by pressure cooker in citrate buffer (pH 6.0 or pH 7.0) before staining with mAb. Tissue sections were incubated with 0.3% H_2_O_2_ in methanol to block endogenous peroxidase activity for 10 min. They were saturated with 10% normal goat serum (Histofine SAB-PO kit; Nichirei Corp) for 30 min at room temperature and then overnight at 4˚C with the primary antibodies in the following dilutions: anti-HLA-HC (clone EMR8-5) 1:1000; anti-β2 microglobulin (clone NAMB-1) 1:100; anti-tapasin (clone TO-3) 1:100; anti-TAP-1 (clone NOB-1) 1:50; anti-TAP-2 (clone NOB-2) 1:400; anti-LMP-7 (clone HB-2) 1:100; and anti-LMP-10 (clone TO-7) 1:200. A biotinylated goat anti-mouse immunoglobulin antibody (Histofine SAB-PO kit; Nichirei Corp) was applied for 30 min at room temperature. Staining was visualized using peroxidase substrate kit 3,3′-diaminobenzidine (Histofine SAB-PO kit; Nichirei Corp). To improve the sharpness of the staining with mAb NOB-1, Target Retrieval Solution, high pH was used (Dako, Kyoto, Japan). Nuclei were lightly counterstained with hematoxylin. TE8 xenografts were used as positive tissue controls. Normal mucosal tissues were used in each specimen as internal controls. LCD xenografts were used as negative tissue controls. Mouse IgG1 was used in place of the primary antibody for negative controls.

Although this study was performed retrospectively, the intensity of cancer tissues compared with normal mucosa lesion in each lesion was evaluated independently by two researchers (K. Tanaka and M. Miyamoto) who were blinded to patient clinical information. A pathologist confirmed the results of these evaluations. The intensity of staining was classified according to a three-level scale: 1, weak staining compared with normal mucosa was observed at more than one spot in the cancer; 2, equivalent staining compared with normal mucosa at all cancer locations; and 3, strong staining compared with normal mucosa at more than one cancer location. When slides were scored by investigators differently (ex: score 1, 2 or score 2, 3), the slides were scored as 2.

### Tissue microarray construction

The archival slides for all cases were reviewed. A slide containing representative tumor was selected, and the total area of tumor was encircled on the slide. The cases that could be cored were selected. Using a manual tissue microarrayer (Alphelys, Plaisir, France), the area needed in the donor block was cored with a 0.6-mm-diameter needle and transferred to a recipient paraffin block. The microarray was constructed with multifold redundancy (four spots in cancer, two spots in normal mucosa for each patient) to increase accuracy. The finalized array blocks were then sliced into 4-μm-thick sections and mounted on glass slides.

### Patients and esophageal specimens

Surgical specimens of resected ESCC that were obtained between March 1994 and November 2004 were used in this study. Patients with primary ESCC underwent radical esophagectomy at the Department of Surgical Oncology, School of Medicine, Hokkaido University. A total of 95 ESCC surgical specimens was examined with the tissue microarray method. Surgical specimens were obtained from 83 males and 12 females (median age 63, range 46–86 years). The median follow-up period was 95.6 months (range 5.1–183.7 months), and 48 patients (50.5%) died during follow-up. No distant organ metastases were detected in any patient on preoperative examinations. Six patients underwent preoperative chemotherapy and/or radiotherapy. Tumor clinicopathologic stage was determined according to the tumor-node-metastasis (TNM) classification system of the International Union Against Cancer (UICC) (31). One of the patients was classified as Tis, 44 as T1, 8 as T2, 38 as T3, and 4 as T4. Overall, 47 patients were classified as N0, and 48 as N1; 75 patients were classified as M0, and 20 as M1. One of the patients was classified as TNM stage 0, 29 as stage I, 8 as stage IIA, 16 as stage IIB, 21 as stage III, 2 as stage IV, 6 as stage IVA, and 12 as stage IVB. All informed consent processes for immunohistochemical staining were conducted in accordance with the guidelines of the Hokkaido University Institutional Review Board authorization for this study.

### Statistical analysis

The chi-square test and Fisher's exact test were used as appropriate. Overall patient survival was calculated from the date of operation to the date of last follow-up or date of patient death. The Kaplan-Meier method was used to estimate overall survival, and survival differences were analyzed by the log-rank test based on APM expression of the cancer lesion compared to normal tissue. The expression level scores were dichotomized by combining scores of 1 and 2 or 2 and 3, depending on which had a more significant relationship with survival on the log-rank test. Univariate and multivariate analyses were performed using the Cox proportional hazard regression model. P<0.05 were regarded as significant in all of the analyses. All analyses were performed with statistical software (Stat-View J version 5.0; SAS Institute Inc., Cary, NC).

## Results

### Expression of HLA class I and APM components in esophageal cell lines on Western blot analysis

**Figure 1:**
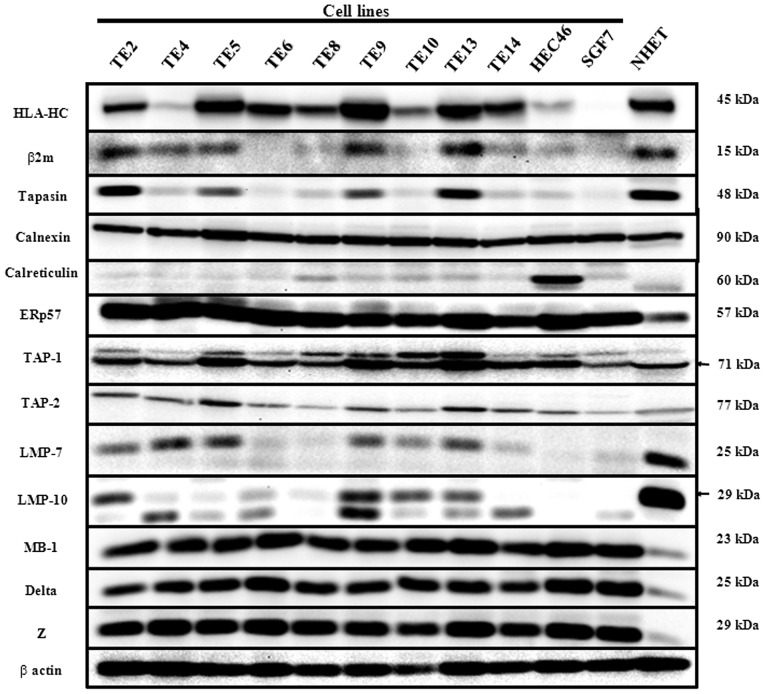
Western blot analysis for HLA class I antigen processing machinery (APM): HLA class I heavy chain (HLA-HC), β2 microglobulin, Tapasin, Calnexin, Calreticulin, ERp57, TAP-1, TAP-2, LMP-7, LMP-10, MB-1, Delta, and Z. Lysates of the cultured human esophageal cancer cell lines (TE2, 4, 5, 6, 8, 9, 10, 13, 14, HEC46, and SGF7) and homogenized normal human esophageal mucosa tissue (NHET) were subjected to Western blot analysis. Lysates of NHET were used as a positive control for APM component. The difference of expression among cell lines was found in seven APM molecules (HLA-HC, β2 microglobulin, Tapasin, TAP-1, TAP-2, LMP-7, LMP-10).

On Western blot analysis with lysates from cultured cell lines, HLA-HC, β2 microglobulin, tapasin, TAP-1, TAP-2, LMP-7, and LMP-10 expressions differed among the cancer cell lines. Most of the cancer cell expressions of these components were downregulated compared with normal human esophageal mucosa tissues (NHET). On the other hand, Calnexin, ERp57, MB1, Delta, and Z did not differ among cultured cell lines. The cancer cell expressions of ERp57, MB1, Delta, and Z were up-regulated compared with NHET. HLA-HC, β2 microglobulin, tapasin, and LMP-7 were synchronously expressed in cell lines such as TE2, TE5, TE9, TE13, HEC46, and SGF7 (Fig. 1). In subsequent experiments, we investigated the components of HLA-HC, β2 microglobulin, tapasin, TAP-1, TAP-2, LMP-7, and LMP-10, which differed among cultured cell lines.

**Figure 2:**
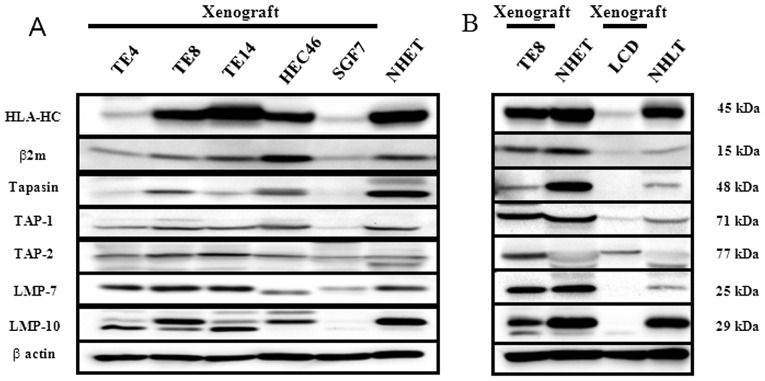
Western blot analysis for the HLA class I APM that had a difference of expression among cell lines with lysates from CB17/severe combined immunodeficiency (SCID) mouse xenograft models. (A) lysates from implanted tumors derived from human esophageal cancer cell lines (TE4, 8, 14, HEC46, and SGF7) and NHET were subjected to Western blot analysis. Each esophageal cancer cell line was implanted subcutaneously into the right flanks of CB17/SCID mice. The expression with lysates from xenografts was almost identical to the expression with lysates from cell lines. (B) Lysates from implanted tumors derived from human esophageal carcinoma cell line (TE8), human lung cancer cell line (LCD), NHET and normal human lung tissue (NHLT) were subjected to Western blot analysis. The expression with lysates from TE8 xenograft was positive in all components, and the expression with lysates from LCD was negative. These xenografts were used to decide the condition of immunohistochemical staining. These experiments were done twice, and the same results were obtained.

The lysates from CB17/SCID xenografts were subjected to Western blot analysis because paraffin blocks of CB17/SCID xenografts were used as positive and negative tissue controls for IHC. Only five cultured esophageal cell lines (TE4, TE8, TE14, HEC46, and SGF7) could be implanted in CB17/SCID mice. Each component's expression in lysates from CB17/SCID xenografts was very similar to that in cultured cell lines. There were few CB17/SCID xenografts of the esophagus with expressions that were negative for every component (Fig. 2A). The lung cell line with negative expression for all components was implanted in CB17/SCID mice. The lysates from implanted tumors derived from TE8, LCD, NHET, and normal human lung tissue (NHLT) were subjected to Western blot analysis (Fig. 2B). There was a human lung cancer cell line (LCD) with negative expression for every component except for TAP2; expression of TE8 was increased in TAP2 compared with LCD.

### Expression of HLA class I and APM components in esophageal cancer patients

**Figure 3:**
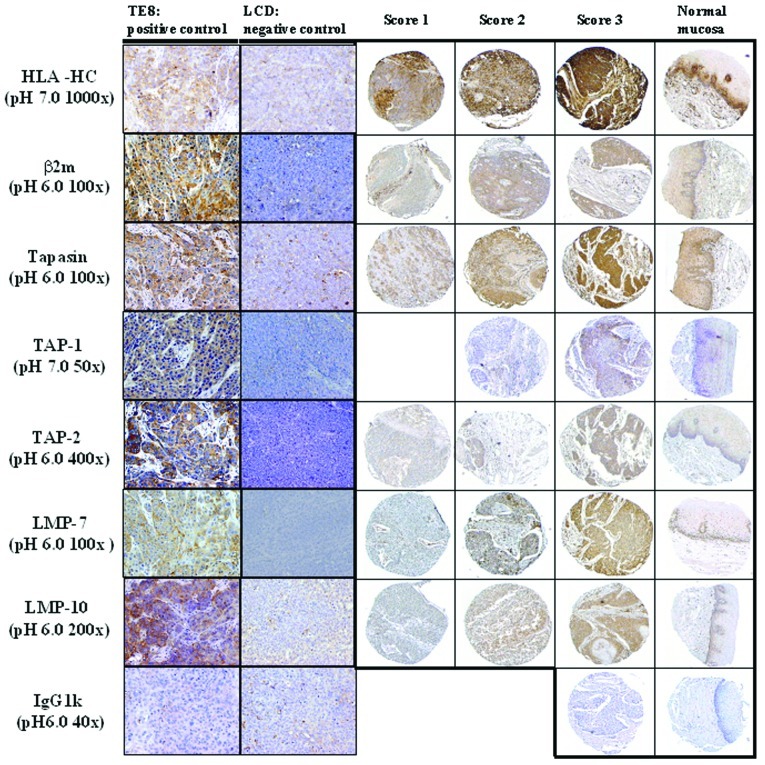
Representative immunohistochemical staining pattern for APM components in xenografts and human esophagus. TE8 xenograft and LCD xenograft, which were positive and negative, respectively, by Western blotting, were stained with mAb (HLA class I heavy chain (HLA-HC), β2 microglobulin, Tapasin, TAP-1, TAP-2, LMP-7, LMP-10). TE8 xenograft was used as a positive control for APM components. LCD xenograft was used as a negative control. The intensity of staining was scored as 1, 2, or 3 indicating weak, clear, or strong expression respectively. Original magnification, ×200 (xenograft), ×100 (carcinoma lesion and normal esophageal mucosa).

On the basis of the results from Western blot analysis, the positive and negative tissue controls for immunostaining were confirmed using CB17/SCID xenografts. As positive controls, normal esophageal mucosa and TE8 xenograft were used. As a negative control, LCD xenograft was used. The conditions of immunohistochemistry that corresponded with the results of Western blotting were identified. Representative immunohistochemical staining patterns for HLA-HC and various APM components are shown for TE8 xenograft, LCD xenograft, carcinoma lesion, and normal esophageal mucosa in Fig. 3. The basement membrane in normal esophageal mucosa was stained with each antibody. HLA-HC, β2 microglobulin, tapasin, TAP-1, and TAP-2 were detected on cell membranes and cytoplasm in both cancer cells and normal mucosa. LMP-7 and LMP-10 were detected in the nucleus and cytoplasm in both cancer cells and normal mucosa (Fig. 3).

Of 95 ESCC specimens, 31 patients (32.6%) were scored as 1, 52 (54.7%) as 2, 12 (12.6%) as 3 for HLA-HC. For β2 microglobulin, 3 patients (3.2%) were scored as 1, 71 (74.7%) as 2, and 21 (22.1%) as 3. For tapasin, 1 patient (1.1%) was scored as 1, 46 (48.4.%) as 2, and 48 (50.5%) as 3. For TAP-1, 73 patients (76.8%) were scored as 2, and 22 (23.2%) as 3. For TAP-2, 4 patients (4.2%) were scored as 1, 73 (76.8.%) as 2, and 18 (18.9%) as 3. For LMP-7, 16 patients (16.8%) were scored as 1, 73 (76.8.%) as 2, and 5 (5.3%) as 3 for LMP-7. For LMP-10, 4 patients (4.2%) were scored as 1, 44 (46.3%) as 2, and 47 (49.5%) as 3.

### Kaplan-Meier survival analysis of HLA class I and APM component expressions

**Figure 4:**
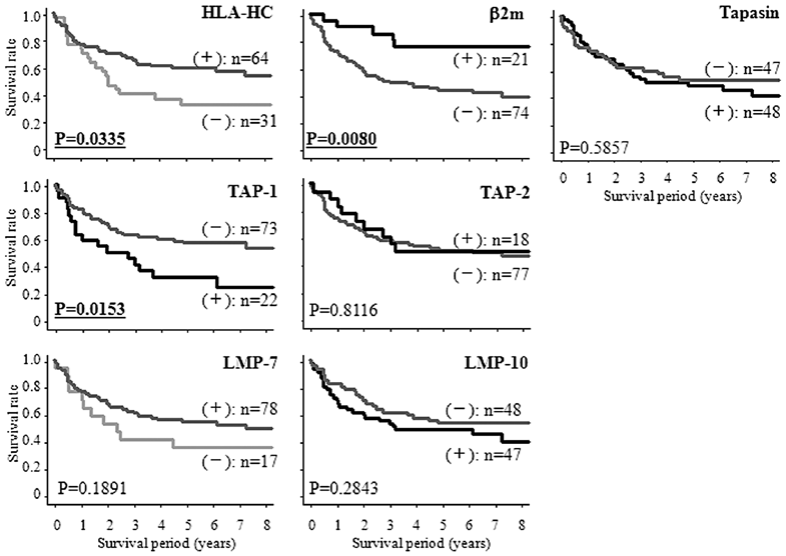
Kaplan-Meier analysis of overall survival according to the expression of APM components. HLA class I heavy chain (HLA-HC) and LMP-7 expression was classified into two groups according to the score (score 1 vs. score 2 and score 3). Score 1 was described as (−). Score 2 and score 3 were described as (+). β2 microglobulin, Tapasin, TAP-1, TAP-2, and LMP-10 expression was classified (score 1 and score 2 vs. score 3). Score 1 and score 2 were described as (−). Score 3 was described as (+). Differences in overall survival were analyzed with the log rank test.

For β2 microglobulin, tapasin, TAP-1, TAP-2, and LMP-10, the patients were divided into two groups according to combining score 1 and score 2 vs. score 3. Scores 1 and 2 were defined as (−), score 3 was defined as (+). For HLA-HC and LMP-7, the patients were divided into two groups according to score 1 vs. combining score 2 and score 3. Score 1 was defined as (−), scores 2 and 3 were defined as (+). The survival rates were significantly lower for patients with down-regulation of HLA-HC than for those without (P=0.0335). The survival rates were significantly lower for patients with up-regulation of TAP-1 than for those without (P=0.0153). The survival rates were significantly higher for patients with up-regulation of β2 microglobulin than for those without (P=0.0080) (Fig. 4).

### Association of HLA class I and APM components with various clinicopathological features

Associations of HLA-HC, β2 microglobulin, and TAP-1 with clinicopathologic features are summarized in Table I. HLA-HC, β2 microglobulin, and TAP-1 expressions were not significantly associated with clinicopathological parameters, such as age, gender, and TNM classification (Table I).

### Univariate and multivariate analyses of HLA class I and APM component expressions and clinicopathologic variables

Univariate analysis for overall survival using a Cox regression model identified HLA-HC (P=0.0081), β2 microglobulin (P=0.0123), TAP-1 (P=0.0177), T classification (P=0.0015), and N classification (P=0.0022) as significant prognostic predictors. Moreover, multivariate analysis of the same set of patients was performed using the significant factors on univariate analysis. The results identified that down-regulation of HLA-HC and up-regulation of TAP-1 were independent unfavorable prognostic factors (hazard ratio, 2.361; P=0.0141 and hazard ratio, 2.297; P=0.0145, respectively). pT classification also had independent prognostic value, with a hazard ratio of 2.341 (P=0.0102) (Table II).

### Analysis of HLA class I and APM component expressions in stage I/II or stage III/IV esophageal cancer patients

Among stage I and II esophageal cancer patients, there were no significant prognostic factors using a Cox regression model. Among stage III and IV esophageal cancer patients, univariate analysis for overall survival using a Cox regression model identified only down-regulation of HLA-HC as a significant prognostic factor (hazard ratio, 2.361; P=0.0138) (Table III).

## Discussion

This study has three major findings. First, expressions of HLA-HC, β2 microglobulin, and five APM components (tapasin, TAP1/2, LMP7/10) were reduced in esophageal cancer cell lines compared with normal tissue, and their components had different expression levels among the esophageal cancer cell lines on Western blot analysis. Second, down-regulation of HLA-HC and up-regulation of TAP-1 were prognostic factors associated with a poor prognosis in patients with esophageal cancer. Overexpression of β2 microglobulin was associated with a good prognosis in patients with esophageal cancer. Third, among stage III and IV cancer patients, HLA-HC down-regulation was an unfavorable prognostic factor, although there was no significant difference between stages I and II.

Western blot analysis showed that most of the components were downregulated, and the expression patterns differed among cell lines. Similar expression patterns of HLA-HC, β2 microglobulin, tapasin, TAP-1, TAP-2, and LMP-7 were observed in TE2, TE5, TE9, TE13, and NHET. These data suggest that the expressions of these components correlate with each other. The factor with a different expression pattern among the cell lines may cause a difference in cell character. By examining these factors and their correlations with clinico-pathologic factors, it might be possible to deduce the role of the factor. Moreover, immunohistochemical staining for these factors would be objective, because both tissue positive controls and negative tissue controls can be easily obtained using these cell lines. These components that were screened by Western blot analysis have been previously reported as prognostic factors in many studies separately (16,22–27). Western blot analysis with cancer cell lines was a useful way to identify candidate prognostic factors.

One of the major characteristics of the present study is the use of CB17/SCID xenografts as positive and negative tissue controls in immunohistochemistry. Based on the expression on Western blotting of CB17/SCID xenografts, the optimal staining conditions were decided to conform the immunohistochemical staining intensity of CB17/SCID xenografts with each antibody (32). By adopting the approach of strict condition setting, the performance of antibodies without nonspecific staining was confirmed. We confirmed that patient specimens could be finely stained under these staining conditions. In our view, making tissue controls is absolutely necessary for immunohistochemical research using antibodies. This is the first report to use the strict approach for choosing the immunohistochemical conditions.

The present study showed that there was a correlation between poor prognosis and HLA-HC down-regulation in esophageal cancer patients. This result was consistent with other reports (16,26). In one of these reports, HLA-HC expression was investigated in ESCC patients using the same antibody (EMR8-5). The frequency of HLA-HC down-regulation and the correlation between prognosis and HLA-HC found in the present study are similar to those described earlier (26). Furthermore, the present results showed that patients with up-regulation of β2 microglobulin had a good prognosis. Many reports showed that down-regulation of β2 microglobulin was associated with a poor prognosis (24,27,33). These reports adopted different immunohistochemistry evaluation criteria from those used in the present evaluation. The other reports adopted two major evaluation methods: one was the percentage of stained tumor cells in an entire lesion (20,24,27), and the other was a combination of percentage and intensity of stained tumor (25,33). There was no category of up-regulation in these two evaluations. The present results also showed that down-regulation of β2 microglobulin was a significant factor related to poor prognosis compared to up-regulation (data not shown).

Surprisingly, in stage III and IV esophageal cancer patients, down-regulation of HLA-HC was a negative prognostic factor. These results support the conjecture that, after radical surgery for advanced cancer, micrometastases of the residual cancer cells expressing HLA class I were eradicated by the immune system. We have already reported that the cooperation between CD4^+^ and CD8^+^ T cells drastically improves the prognosis of patients with ESCC (5). Our results support that the host immune response against cancer cells and the status of HLA class I molecules on cancer cells are important factors for postoperative prognosis. If the cancer cells in surgical specimens express HLA-HC, the patients would undergo adjuvant immunotherapy to eradicate residual tumor. It may be possible to change the postoperative adjuvant therapy based on HLA-HC expression.

In conclusion, the current results suggest that three HLA class I APMs (HLA-HC, β2 microglobulin, and TAP-1) are correlated with postoperative survival in esophageal cancer, and that the down-regulation of HLA-HC in tumors is associated with a poor prognosis among stages III and IV esophageal cancer patients. Thus, the present study suggests that immunohistochemical staining for HLA-HC is useful as a prognostic marker and for selection of adjuvant therapy in stage III and IV esophageal cancer patients.

## Figures and Tables

**Table I:** Correlation between clinicopathological features of the 95 ESCC patients and expression of APM components.

	HLA class I Heavy Chain			β2-microglobulin			TAP-1		
			
	(−) n=31	(+) n=64	P-value	(−) n=74	(+) n=21	P-value	(−) n=73	(+) n=22	P-value
Age (years)									
≥63	20	31	0.141	40	11	0.892	43	8	0.063
<63	11	33		34	10		30	14	
Gender									
Male	28	55	0.745^a^	67	16	0.129^a^	63	20	0.727^a^
Female	3	9		7	5		10	2	
pT classification									
T1	15	29	0.890	32	12	0.131	35	9	0.489
T2/T3/T4	16	34		42	8		37	13	
pN classification									
Negative	18	29	0.244	33	14	0.074	39	8	0.161
Positive	13	35		41	7		34	14	
pM classification									
M0	26	49	0.413	59	16	0.765^a^	58	17	0.775^a^
M1	5	15		15	5		15	5	
P-stage									
0/I/II	18	36	0.867	40	14	0.3030	43	11	0.460
III/IV	13	28		34	7		30	11	

a Fisher's exact test was used.

**Table II:** Univariate and multivariate analyses of patients.

	Univariate			Multivariate		
		
	HR	95% CI	P	HR	95% CI	P
HLA-HC						
−/+	1.855	1.040–3.309	**0.0081**	2.361	1.189–4.686	**0.0141**
β2m						
−/+	3.271	1.294–8.269	**0.0123**	2.006	0.750–5.361	0.1652
Tapasin						
+/−	1.171	0.663–2.067	0.5861			
TAP-1						
+/−	2.073	1.135–3.786	**0.0177**	2.297	1.179–4.476	**0.0145**
TAP-2						
+/−	1.092	0.529–2.256	0.8117			
LMP-7						
−/+	1.566	0.797–3.077	0.1928			
LMP-10						
+/−	1.362	0.722–2.405	0.2862			
Age (years)						
≥63/<63	1.307	0.736–2.321	0.3600			
Gender						
Male/Female	1.871	0.671–5.214	0.2311			
pT classification						
T2T3T4/T1	2.661	1.456–4.868	**0.0015**	2.341	1.224–4.479	**0.0102**
pN classification						
1/0	2.441	1.087–3.471	**0.0022**	1.393	0.723–2.685	0.3214
pM classification						
M1/M0	1.587	0.824–3.056	0.1672			
P-Stage						
III IV/I II	2.907	1.628–5.189	0.0003			

HR, hazard ratio; 95% CI, 95% confidence interval; HLA-HC, HLA class I heavy chain; β2m, β2-microglobulin. Bold, statistically significant.

**Table III:** Univariate analyses of patients in stage I/II and stage III/IV.

	Stage I/II			Stage III/IV		
		
	HR	95% CI	P	HR	95% CI	P
HLA-HC						
−/+	1.521	0.619–3.739	0.3609	2.751	1.230–6.154	**0.0138**
β2m						
−/+	2.317	0.678–7.913	0.1799	4.004	0.946–16.943	0.0594
Tapasin						
−/+	0.604	0.250–1.461	0.2635	1.567	0.742–3.309	0.2389
TAP-1						
+/−	1.986	0.761–5.183	0.1607	1.987	0.915–4.316	0.0828
TAP-2						
−/+	0.710	0.257–1.958	0.5081	1.565	0.542–4.522	0.4081
LMP-7						
−/+	1.581	0.573–4.366	0.3763	1.723	0.681–4.356	0.2504
LMP-10						
+/−	1.068	0.442–2.580	0.8832	1.367	0.639–2.926	0.4200
Age (years)						
≥63/<63	1.254	0.519–3.029	0.6143	1.164	0.544–2.490	0.6949
Gender						
Male/Female	3.548	0.473–26.596	0.2179	1.244	0.375–4.131	0.7213
pT classification						
2,3,4/1	2.117	0.842–5.319	0.1107	-	-	-
4/1,2,3	-	-	-	1.702	0.403–7.184	0.4689
pN classification						
1/0	1.345	0.536–3.376	0.5272	1.185	0.480–2.928	0.7123
pM classification						
0/1	-	-	-	1.473	0.695–3.123	0.3121
pStage						
2/1	1.358	0.565–3.268	0.4940	-	-	-
4/3	-	-	-	1.473	0.695–3.123	0.3121

HR, hazard ratio; 95% CI, 95% confidence interval; HLA-HC, HLA class I heavy chain; β2m, β2-microglobulin. Bold, statistically significant.
